# Culture modulates face scanning during dyadic social interactions

**DOI:** 10.1038/s41598-020-58802-0

**Published:** 2020-02-06

**Authors:** Jennifer X. Haensel, Matthew Danvers, Mitsuhiko Ishikawa, Shoji Itakura, Raffaele Tucciarelli, Tim J. Smith, Atsushi Senju

**Affiliations:** 1 Birkbeck, University of London, Department of Psychological Sciences, London, WC1E 7HX United Kingdom; 2 Kyoto University, Department of Psychology, Kyoto, 606-8501 Japan

**Keywords:** Social behaviour, Psychology, Human behaviour

## Abstract

Recent studies have revealed significant cultural modulations on face scanning strategies, thereby challenging the notion of universality in face perception. Current findings are based on screen-based paradigms, which offer high degrees of experimental control, but lack critical characteristics common to social interactions (e.g., social presence, dynamic visual saliency), and complementary approaches are required. The current study used head-mounted eye tracking techniques to investigate the visual strategies for face scanning in British/Irish (in the UK) and Japanese adults (in Japan) who were engaged in dyadic social interactions with a local research assistant. We developed novel computational data pre-processing tools and data-driven analysis techniques based on Monte Carlo permutation testing. The results revealed significant cultural differences in face scanning during social interactions for the first time, with British/Irish participants showing increased mouth scanning and the Japanese group engaging in greater eye and central face looking. Both cultural groups further showed more face orienting during periods of listening relative to speaking, and during the introduction task compared to a storytelling game, thereby replicating previous studies testing Western populations. Altogether, these findings point to the significant role of postnatal social experience in specialised face perception and highlight the adaptive nature of the face processing system.

## Introduction

Although behavioural, cognitive, and neural processes underlying face perception have typically been assumed to be universal^1^, eye tracking studies have recently identified significant cultural modulations on visual strategies during face perception tasks. When asked to recognise face identities with neutral expressions presented statically on screen, Western Caucasians exhibited a greater triangular scanning pattern of the eyes and mouth, whereas East Asians showed more fixations on the nose^2–6^. A proposed explanation is based on culture-specific attentional styles of holistic versus analytic perception^7–9^, with Western Caucasians fixating critical facial features directly, and East Asians using their extrafoveal vision more effectively to extract visual information by fixating the nose^10–13^. When categorising^14^ or free-viewing emotionally expressive faces^15^, Western Caucasians scanned the mouth region significantly more, whereas East Asians fixated the eye region. This may reflect an adjustment to the culture-specific patterns of emotional expressions of faces, with East Asians representing the intensity of emotional expressions with movements of the eyes and Western Caucasians using other face regions^16^. These studies thus demonstrated cultural modulations during face processing, and the precise manifestation of eye movement patterns may differ depending on stimulus characteristics or task demand.

## Limitations of Screen-Based Paradigms

To date, cross-cultural comparisons on face scanning have been restricted to screen-based paradigms and largely employed static images of faces. Such highly controlled experiments have the advantage of isolating and manipulating specific factors underlying face scanning strategies to reveal detailed insights that can explain or generate precise hypotheses on cultural differences. However, such controlled experiments can also considerably limit interpretations on social attention when ultimately the goal is to understand ‘real-world’ behaviour. First, static images displayed on a screen do not contain dynamic properties; however, low-level motion can predict gaze locations^17^, raising the possibility that more salient facial features (e.g., a moving mouth during speech) could modulate face scanning. Moreover, participants in screen-based paradigms cannot typically interact with the viewed face, and the lack of social presence implies that sociocultural norms are not considered. Visual orienting to faces, for instance, has been shown to decrease significantly when participants sat in the same room with another person, compared to when participants saw a videotape of the same individual^18^, highlighting the importance of social presence in naturalistic settings. Furthermore, cultural differences have been found when faces on screen appeared to make direct eye contact with the participant, with East Asians scanning the nose and Western Caucasians fixating the eyes^19^. No cultural differences were observed, however, when the faces appeared to avert their gaze, thereby pointing to possible cultural differences in eye contact avoidance^19^, which has been proposed to act as a sign of respect in East Asian cultures^20,21^. To examine cultural modulations on naturalistic face scanning, studies will therefore need to take into account such sociocultural norms. Participants in screen-based studies are additionally instructed to look at the screen throughout the testing session. Given that only the face image typically appears on screen, these paradigms cannot examine the extent to which individuals visually orient to faces spontaneously within naturalistic social contexts, which are typically characterised by environmental visual distractors. Although previous face-to-face interaction studies have revealed decreased face looking when participants were speaking compared to when they were listening^22^ – suggesting that contextual factors inherent to dyadic interactions can modulate face orienting behaviour – these studies were conducted in Western cultures and no study to date has examined whether naturalistic face orienting may differ between cultures.

## The Current Study

This study aimed to provide first evidence of cultural differences in face scanning within naturalistic dyadic interactions. Eye movements of British or Irish and Japanese adults were recorded using head-mounted eye tracking techniques while participants interacted with a local research assistant (in the UK or in Japan); both the participant and the local research assistant introduced themselves and played a storytelling game. Since previous face-to-face interaction studies^22^ (conducted in Western cultures) revealed significantly greater face looking during listening compared to speaking periods, we also included speech states as a factor in the present cross-cultural study, and expected to replicate findings in both cultural groups. Given the assumption that the local research assistants would exhibit facial expressions of emotion (e.g., smiling), we predicted cultural differences to converge with screen-based findings using emotionally expressive face stimuli^14,15^. Specifically, Japanese participants would scan the eye region proportionally more, whereas British/Irish individuals would exhibit increased mouth looking. In line with previous face-to-face interaction paradigms that recorded autistic and social anxiety traits^23–25^, and screen-based studies pointing to a possible relationship between higher social anxiety and reduced face and gaze scanning^26,27^, the Autism Quotient (AQ)^28^ and self-report version of the Liebowitz Social Anxiety Scale (LSAS-SR)^29^ were administered to ensure that any cultural differences in face orienting or scanning were not solely driven by differences in autistic or social anxiety traits.

Two methodological innovations were implemented to achieve the aim of this study. First, we developed a semi-automatic gaze classification method that combined rapid automatic face detection and tracking algorithms with a manual moderation stage to allow user interference in case automatic processes fail. A gaze classification method was required since scene recordings obtained from head-mounted eye trackers differ between individuals with the face changing size, position, and angle in every frame – the location of the conversational partner’s face can therefore not be known *a priori*. The present two-stage gaze classification method also provided a rapid approach compared to common manual coding procedures, which are highly time-consuming and often require multiple coders given that gaze data needs to be annotated on a participant-by-participant and frame-by-frame basis^30,31^. The addition of a manual moderation stage furthermore improved gaze classification accuracy compared to fully automatic methods, which often result in insufficient accuracy rates due to the low-quality nature of scene recordings. The output of this gaze classification method was used to examine face scanning; specifically, traditional regions-of-interest (ROI) analyses were conducted to investigate scanning of the upper and lower face regions as a proxy for the eyes and mouth, respectively.

Secondly, we complemented the ROI approach with novel, spatially sensitive data-driven analyses based on Monte Carlo permutation testing, thereby making no *a priori* assumptions about which face regions should be included in the analysis to examine cultural differences in face scanning^32^. Although ROI analyses are statistically sensitive to detecting differences in eye movement behaviour between groups or conditions due to the spatial pooling of gaze data, restricting analyses to the upper and lower face may hinder new insights. Compared to Western Caucasians, for instance, East Asians have been found to exhibit increased nose scanning during screen-based recognition tasks using neutral face stimuli^2^. This could not be revealed with ROI analyses given that the nose region would fall onto the boundary between the upper and lower face. Eye movements may additionally manifest differently within naturalistic social contexts. A data-driven approach would thus capture cultural differences which could otherwise be missed when using traditional ROI analyses.

## Results

### Regions-of-interest analysis

Fixations were extracted using default settings in *BeGaze* (Version 3.7; SensoMotoric Instruments, Germany) with a minimum duration of 50 ms and a maximum dispersion of 0.5°. We developed semi-automatic tools implemented in MatLab (R2015a, MathWorks; see Supplementary Methods for a detailed description) to locate the upper and lower face regions and to classify fixations accordingly.

Two key dependent variables were extracted (see Supplementary Data). First, face fixation time was calculated proportional to valid total fixation duration (with a cut-off at 30 s per task). Periods of data loss (e.g., blinks, track loss) were excluded from the valid time to consider only those periods during which it was possible to confidently state whether or not participants engaged in face looking. Data loss did not significantly differ between groups (Japanese: *M* = 9.60%, *SD* = 7.25%; British/Irish: *M* = 6.62%; *SD* = 5.15%; *t*(54) = 1.78, *p* = 0.080). Secondly, upper face fixation time, which served as a proxy for fixation duration on the eyes, was computed proportional to face fixation time.

A 2 (Group: British/Irish, Japanese) × 2 (Speech: speaking, listening) × 2 (Task: introduction, storytelling) mixed ANOVA was conducted on fixation time, separately for proportional face and upper face looking. Although we had no specified predictions with respect to the experimental tasks (introduction and storytelling), we included the factor Task in an exploratory manner. Shapiro-Wilk Tests suggested that the assumption of normality was not always met (*p* < 0.05) and this could not be corrected with data transformations. Given that no equivalent non-parametric version exists, the 2 × 2 × 2 mixed ANOVA was conducted and significant effects were followed up or confirmed using appropriate non-parametric tests.

For proportional face fixation time, a significant main effect of Speech was revealed (*F*(1,54) = 182.95, *p* < 0.001, *ƞ*_*p*_^*2*^ = 0.772; confirmed using the Wilcoxon Signed-Rank Test: *Z* = 6.42, *p* < 0.001, *r* = −0.607), with both cultural groups fixating the face of the research assistant more during periods of listening than speaking (Table 1). Face looking was also significantly greater for the introduction task relative to the storytelling game (*F*(1,54) = 25.56, *p* < 0.001, *ƞ*_*p*_^*2*^ = 0.321; confirmed using Wilcoxon Signed-Rank Test: *Z* = −4.10, *p* < 0.001, *r* = 0.388). A significant Speech × Group interaction was also found (*F*(1,54) = 4.83, *p* = 0.032, *ƞ*_*p*_^*2*^ = 0.082); however, post-hoc comparisons using the non-parametric Mann Whitney U test showed that the speaking and listening conditions did not differ between the two groups (speaking: *U* = 287, *p* = 0.087; listening: *U* = 364, *p* = 0.652). There were no other significant main effects or interactions (Group: *F*(1,54) = 0.09, *p* = 0.770; Task × Group: *F*(1,54) = 0.43, *p* = 0.513; Speech × Task: *F*(1,54) = 3.99, *p* = 0.051; Speech × Task × Group: *F*(1,54) = 1.04, *p* = 0.312). In other words, cultural modulations on proportional face fixation time could not be observed.

**Table 1:** Medians and interquartile ranges for face fixation time (in %).

		Japanese *Mdn (IQR)*	British/Irish *Mdn (IQR)*
Introduction	Speaking	43.95 (32.76)	63.89 (30.32)
	Listening	84.14 (18.64)	91.02 (22.28)
Storytelling	Speaking	31.05 (34.79)	39.77 (42.79)
	Listening	81.46 (19.54)	80.00 (24.79)

For upper face fixation time, Japanese participants spent a greater proportion of overall face fixation time scanning the upper face region, compared to the British/Irish group (Table 2; Group: *F*(1,54) = 10.47, *p* = 0.002, *ƞ*_*p*_^*2*^ = 0.162; confirmed using Mann Whitney U test: *U* = 248, *p* = 0.019, *r* = −0.314), but no other main effects or interactions were found (Speech: *F*(1,54) = 0.10, *p* = 0.759; Task: *F*(1,54) = 3.52, *p* = 0.066; Speech × Group: *F*(1,54) = 1.83, *p* = 0.182; Task × Group: *F*(1,54) = 0.01, *p* = 0.946; Speech × Task: *F*(1,54) = 0.89, *p* = 0.349; Speech × Task × Group: *F*(1,54) = 0.04, *p* = 0.853). In other words, cultural modulations were observed, but other contextual factors such as speech or task did not influence proportional upper face fixation time.

**Table 2:** Medians and interquartile ranges for upper face fixation time (in %).

		Japanese *Mdn (IQR)*	British/Irish *Mdn (IQR)*
Introduction	Speaking	79.40 (31.72)	58.71 (61.12)
	Listening	84.66 (25.53)	53.10 (67.12)
Storytelling	Speaking	69.33 (42.66)	57.70 (46.03)
	Listening	78.25 (32.86)	49.26 (56.62)

### Monte Carlo permutation test

To examine face scanning in a spatially sensitive manner, we adopted data-driven analysis methods based on Monte Carlo permutation testing (see Supplementary Methods for a more detailed description). First, all gaze points that fell within the face region were re-mapped into a unified coordinate system that represented the face region (see Supplementary Methods), and collapsed across time to produce gaze density maps. Given that statistically contrasting the gaze density maps between cultural groups introduces the *multiple comparison problem*, adjustment of the alpha-level from a local scale (i.e., a single pixel) to a global scale (i.e., the entire map) is required. The Bonferroni correction method assumes independence between pixels and approximates an adjusted significance threshold by dividing the value of the alpha-level by the number of tests. However, smoothing of eye movement data results in spatial dependency of gaze points, making the Bonferroni approach overly conservative. An alternative method is based on Random Field Theory (RFT)^33,34^, which also provided the framework for *iMap*^35^. RFT requires a Gaussian distribution and sufficient smoothness, and represents a powerful method when assumptions are met. However, methods based on RFT may produce unreliable results when data is not normally distributed or for paradigms with a low number of participants since maps may not necessarily be sufficiently smooth^36^. Another approach – and the one chosen here – is non-parametric permutation testing^37–39^. Permutation testing uses the observed data itself to generate a null distribution by exchanging the data across groups in all possible arrangements to compute the frequency distribution of test statistics (e.g., *t-*score). Given that computing all possible permutations is time-consuming and computationally demanding, the *Monte Carlo method*^40^ can approximate the null distribution by running permutations in the order of several thousand iterations. We conducted *cluster-based* permutation tests, whereby a clustering procedure was applied to the original data set and to each permuted data set. The clustering procedure involved identifying neighbouring pixels if their test criterion was greater than the critical value *t*_crit_ associated with a specified *p*-value threshold (here 0.01). To examine which clusters in the original map were significant, a cluster statistic was selected and used as comparison with each permuted map (here the statistic was the size of the cluster). After all iterations were performed (here the number of iterations was set to 10,000), the proportion of test statistics that resulted in a larger value than the observed statistic was obtained and flagged as significant if this occurred less than 5% (0.05) of times.

**Figure 1:**
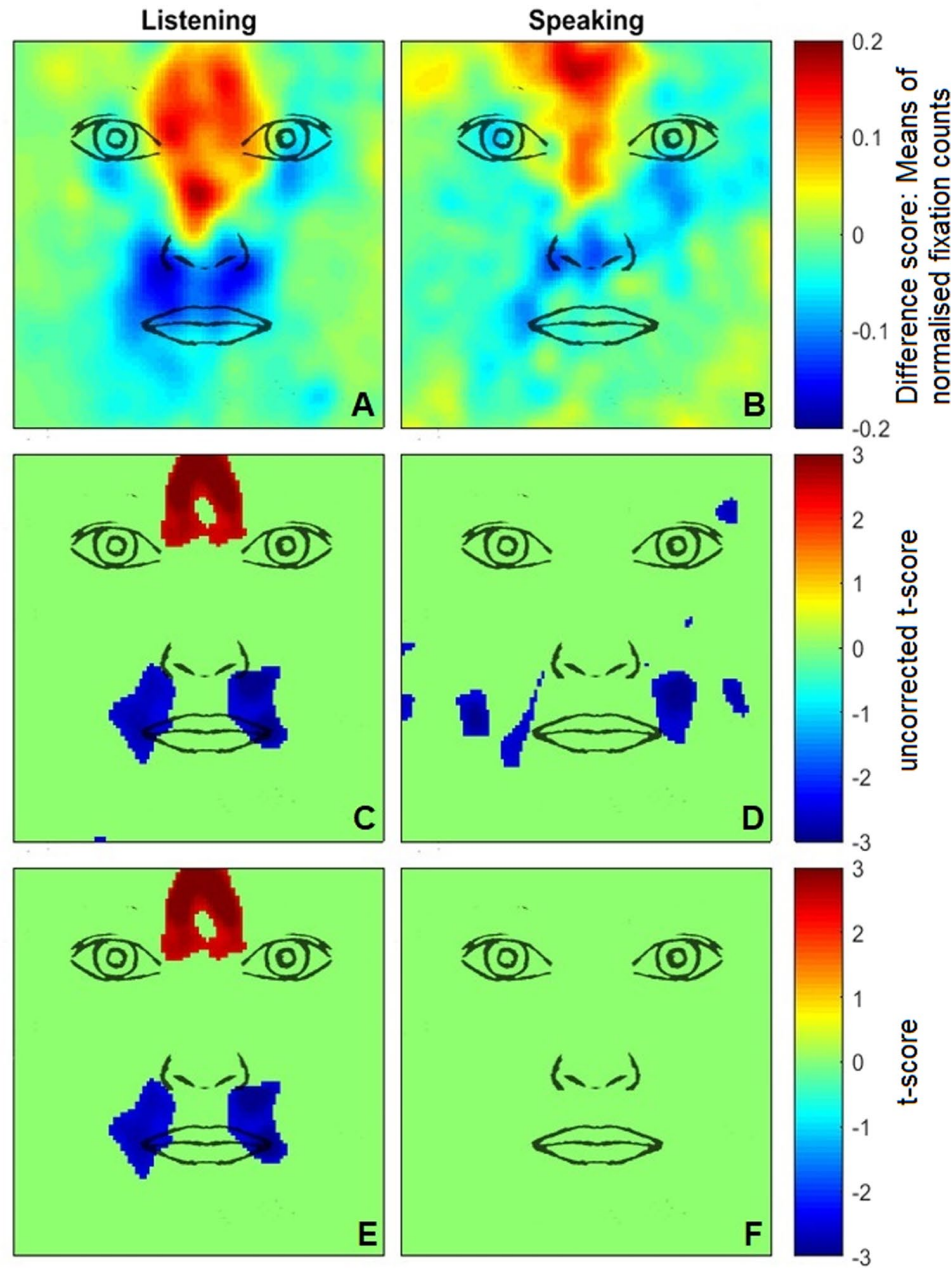
Descriptive and statistical gaze density difference maps. Red and blue regions indicate significantly greater scanning in Japanese and British/Irish participants, respectively. (**A**) Descriptive difference map for face scanning during periods of listening and (**B**) during periods of speaking. (**C**) Uncorrected *t*-scores (*p* < 0.01) indicate several gaze clusters for periods of listening, (**D**) as well as periods of speaking. (**E**) Monte Carlo permutation testing revealed significant gaze clusters for periods of listening, (**F**) but not for periods of speaking.

The Monte Carlo permutation test was implemented in MatLab (R2015a, MathWorks) using the *CoSMoMVPA* toolbox^41^ and *FieldTrip* toolbox^42^. The present analysis collapsed fixation data across the introduction task and storytelling game, and was performed separately for speaking and listening periods. The statistical analysis revealed significant clusters for listening but not speaking periods (Fig. 1). When listening, Japanese participants scanned the region between the eyes more than the British/Irish group, in line with the ROI findings. British/Irish individuals, meanwhile, scanned the mouth region more than the Japanese group (see Supplementary Data for density maps and analysis scripts).

### AQ and LSAS scores

British/Irish participants obtained a significantly lower AQ score (*M* = 14.00, *SD* = 6.28, ranging from 5 to 30) than Japanese individuals (*M* = 21.59, *SD* = 8.65, ranging from 10 to 39; *t*(54) = 3.78, *p* < 0.001, *d* = 1.00; see also Supplementary Data). Spearman correlations were conducted separately for each cultural group to examine any potential relationship between AQ scores and fixation time on the (upper) face. For face looking, the analysis was conducted separately for the introduction task and storytelling game given the task effects identified by the ROI analysis. Results showed no significant correlations, except for a negative correlation between AQ scores and upper face fixation time during periods of speaking in the British/Irish group (*r*_s_(29) = −0.431, *p* = 0.020; Table 3). In other words, increased autistic traits were associated with decreased upper face scanning only in British/Irish participants and only when they were speaking. British/Irish participants also had a significantly lower LSAS score (*M* = 36.45, *SD* = 16.32, ranging from 10 to 75) than Japanese individuals (*M* = 48.19, *SD* = 21.07, ranging from 10 to 94; *t*(54) = 2.34, *p* = 0.023, *d* = 0.62). No significant correlations were revealed, except for a negative correlation between LSAS scores and face fixation time for the introduction task during periods of speaking in the Japanese group (*r*_s_(27) = −0.480, *p* = 0.011; Table 3). In other words, greater social anxiety traits were associated with decreased face looking only in the Japanese group and only in a specific task and speech condition.

**Table 3:** Spearman’s rho and corresponding *p-*values for the relationship between AQ/LSAS scores and (upper) face fixation time.

		Japanese		British/Irish	
		AQ	LSAS	AQ	LSAS
Speaking	Face (Intro)	−0.268 *p* = 0.177	−0.480* *p* = 0.011	−0.101 *p* = 0.602	−0.244 *p* = 0.202
	Face (Story)	−0.277 *p* = 0.162	−0.078 *p* = 0.698	−0.071 *p* = 0.715	−0.312 *p* = 0.099
	Upper face	−0.171 *p* = 0.393	−0.312 *p* = 0.113	−0.431* *p* = 0.020	0.134 *p* = 0.489
Listening	Face (Intro)	0.001 *p* = 0.996	−0.025 *p* = 0.901	−0.119 *p* = 0.538	−0.326 *p* = 0.085
	Face (Story)	−0.310 *p* = 0.115	−0.207 *p* = 0.301	−0.004 *p* = 0.983	−0.008 *p* = 0.968
	Upper face	0.221 *p* = 0.267	0.067 *p* = 0.741	−0.195 *p* = 0.312	−0.067 *p* = 0.729

*p < 0.05.

## Discussion

The current study aimed to examine cultural differences in face scanning during dyadic social interactions. The ROI analysis revealed greater face orienting during periods of listening compared to speaking, replicating findings from previous studies^22^ and suggesting a robust speech effect across different face-to-face interaction paradigms. Decreased face orienting when speaking may reflect a tendency for individuals to avert their gaze during more cognitively demanding periods to reduce cognitive load^43^. Meanwhile, increased face looking when listening could have helped participants to decode speech^44^, and may have also functioned as a social signal to indicate to the conversational partner that one is still listening^45^. Furthermore, greater face orienting was observed for the introduction task compared to the storytelling game. This task effect could reflect greater social signalling when dyads were less familiar with each other, or indicate a need to avert gaze in order to reduce cognitive load given the more demanding nature of the storytelling game^46^, which required participants to recall and describe a past event or experience.

Both the ROI and permutation analysis revealed cultural differences in face scanning. Japanese participants directed a significantly greater proportion of face looking time to the upper region compared to British/Irish individuals, mirroring screen-based findings employing emotionally expressive face stimuli^14,15^. The increased upper face scanning observed in the Japanese group thus appears to contradict the eye contact avoidance theory, which postulates less mutual gaze in East Asian populations^20^. Alternatively, Japanese participants could have directed their gaze *between* the eyes – as indicated by the results from the permutation analysis – which would be consistent with both the present ROI findings as well as the eye contact avoidance theory. Given that the current study did not record eye movements of both individuals in each dyad, future studies will be required to examine cultural differences in eye contact in more detail.

The observed cultural differences in upper face scanning may reflect greater social signalling in Japanese compared to British/Irish individuals. It may have also been visually more informative for Japanese participants to scan the upper face region. East Asians have previously been shown to fixate the eye region when categorising emotional expressions, whereas Western Caucasians exhibited greater gaze distribution across the face^14^. Given that the research assistants showed emotional facial expressions (e.g., smiling), this could account for the increased upper face scanning in the Japanese group. Future cross-cultural studies could attempt to disentangle the effects of social presence and the role of visual information by recording the conversational partner and tracking eye movements as participants watch the video footage^22^. Conversely, increased attention to the mouth region could have been more visually informative for British/Irish participants. While both Japanese and English native speakers benefit from attending to the mouth when decoding speech^44^, phonological differences between the English and Japanese languages could contribute to cultural differences in audio-visual speech perception and in turn cultural differences in face scanning. Lip-read information has been shown to be less ambiguous and therefore more informative in English compared to Japanese^47^; for instance, English consonants can be categorised into a higher number of consonant groups by lip-reading than Japanese consonants^48^. This is also consistent with findings demonstrating that additional visual cues do not benefit Japanese second language learners in consonant perception^49^, and that Japanese individuals exhibit a significantly reduced McGurk effect^50^. It may therefore have been less informative for Japanese participants to look at the mouth, resulting in the observed cultural differences in face scanning.

Findings from the permutation analysis additionally showed that only the listening but not the speaking condition gave rise to cultural differences in face scanning. Quantitative checks of the gaze density maps revealed similar variance of the data distribution for the listening and speaking condition, suggesting that the difference in the findings from the permutation analysis were unlikely due to differences in variance. Since both cultural groups engaged in significantly less face orienting when speaking than when listening, the null finding could also have resulted from insufficient eye movement data. This is supported by the descriptive heat maps (Fig. 1), which were characterised by similar density patterns, but the speaking condition showed weaker effects.

The current study also included AQ and LSAS measures to investigate whether face scanning was associated with autistic or social anxiety traits. Japanese participants exhibited significantly higher autistic and social anxiety traits than British/Irish individuals, in line with previous findings^25,51–53^. For AQ scores, a significant correlation was only found between increased autistic traits and decreased upper face scanning during periods of speaking. This was only observed in the British/Irish group, but not for Japanese participants, suggesting that cultural differences in upper face scanning cannot simply be explained by cultural differences in autistic traits. Crucially, however, the significant correlation was only observed for periods of speaking, whereas cultural differences in upper face scanning were also revealed for periods of listening, indicating that autistic traits unlikely fully explained the observed cultural differences. Furthermore, we found a significant correlation between greater social anxiety traits and decreased face orienting in the introduction task for Japanese participants when they were speaking. Decreased face orienting may have been considered more socially appropriate during speaking periods (e.g., gaze aversion when listening to another person could signal disinterest), thereby allowing participants to look at the face at a degree with which they were comfortable. Crucially, social anxiety traits also unlikely fully explained the present cultural differences, given that significant correlations were not observed for upper face fixation time, which was the dependent variable that revealed cultural differences.

The nature of face-to-face interaction paradigms and associated cross-cultural comparisons are inherently characterised by shortcomings that need to be acknowledged. Unlike screen-based paradigms, which can more easily attribute the source of experimental effects to the face stimuli, the participant, or both, the speaker’s behaviour within a dyadic interaction is never entirely independent from the listener’s behaviour (and vice versa). Given this interdependency, the source of culture-specific scanning patterns cannot be determined in a straightforward manner. It is thus possible that the observed face scanning strategies were specific to the local research assistant. Unlike the Japanese research assistant, for instance, the beard of the British research assistant could have elicited greater mouth looking in British/Irish participants. Given that the increased scanning of the lower face was restricted to the area immediately surrounding the mouth region, the lack of eye movements directed toward the remaining lower face region suggests that participants may have focused on the mouth region *per se*. It is possible though that British/Irish participants showed increased scanning of the mouth since the beard may have made it more difficult to decode the research assistant’s lip movements (i.e., speech). However, this account should then not hold for periods of listening, but the ROI findings demonstrated that increased mouth scanning was not dependent on speaking and listening periods. Matching research assistants across cultures and ethnicities is practically impossible, and any attempt to experimentally manipulate natural culture-specific behaviours may mask dynamic characteristics of social interactions that would otherwise give rise to true cultural differences in face scanning. To minimise the possibility that findings were unique to the interacting individual, several research assistants could alternatively be employed for each cultural group, or virtual characters could be used to allow for greater experimental control; however, this also comes with practical and resource challenges, and would further increase data variability. To rule out the possibility that the observed cultural differences in the current study were solely driven by idiosyncratic behaviours of the local research assistants, we analysed a subset of eye movement data from a separate screen-based face scanning study that tested the majority of the current participants^54^ (see Supplementary Analysis for details). The screen-based data was included here as supportive evidence showing that idiosyncratic behaviours *per se* unlikely explained the cultural differences in face scanning observed in the present dyadic interactions, rather than as a direct quantitative comparison with the data from the current study. Cultural differences in scanning patterns were observed for faces displayed as static images (e.g., more mouth scanning in the British group), and dynamic videos (e.g., increased nose scanning in Japanese participants), using face identities of both White-British and Japanese ethnicities. The precise manifestation of scanning patterns varied between the screen-based experiment and the present dyadic interaction study, which could be attributed to various methodological factors (e.g., greater low-level saliency in the eye region for the current study), the considerably weaker influence of sociocultural norms in screen-based paradigms (e.g., no social signalling), and context- and stimulus-dependent differences in visual information use for face processing^55^. For instance, visually informative regions of dynamic faces with neutral expressions (as in the screen-based study) may be more spatially distributed across the face, with East Asians deploying central face scanning strategies to extract information from multiple facial features^10–13^. In contrast, visually informative regions for more expressive faces (as in the current study) may be largely contained in the eye region for East Asians^16^, who in turn fixate the eyes directly. Crucially, the Supplementary Analysis suggests that the cultural differences observed in the present dyadic interactions were unlikely entirely driven by the local research assistant’s idiosyncratic behaviour since the cultural groups also differed in their scanning patterns when presented with other face identities.

In sum, we applied a semi-automatic gaze classification method to conduct traditional ROI analyses and employed a complementary data-driven approach to allow for a more refined description of cultural modulations on face scanning during dyadic interactions. Factors including speech (listening versus speaking) and task demand influenced how often participants gazed at the face, highlighting the context-dependent nature of face orienting. Crucially, Japanese participants gazed more at the eye and central face region while British/Irish individuals looked at the mouth, demonstrating cultural modulations on face scanning during dyadic interactions for the first time. The current study also provides a methodological framework for precisely studying cultural influences on gaze behaviour during dyadic social interactions, and for informing future studies aiming to examine eye movement behaviour in screen-based versus ‘real-world’ scenarios. Altogether, the present findings point to the important role of postnatal social experiences in the visual strategies used for specialised face perception, highlighting the adaptive nature of the face processing system.

## Method

### Participants

Thirty-six British/Irish and 34 Japanese adults participated in this study. British/Irish participants were tested at Birkbeck, University of London (UK), and Japanese participants were tested at Kyoto University (Japan). Seven British/Irish participants were excluded due to flickering gaze data (*N* = 6) or because the face of the research assistant was not captured (*N* = 1; this occurs when the participant’s head is tilted downward). Seven Japanese participants were also excluded from analysis due to flicker (*N* = 6) or because the participant had previously lived in Western Europe (*N* = 1). The drop-out rate (19.4% and 20.6% for British/Irish and Japanese participants, respectively) was higher than in typical screen-based eye tracking experiments, largely due to participants smiling during the social interaction which occluded the pupil or corneal reflections. The final sample for analysis therefore included 29 British/Irish (13 female) and 27 Japanese (14 female) adults.

British/Irish participants (*M* = 28.07 years, *SD* = 6.60 years) were born and raised in the UK or Republic of Ireland, were of White-British or White-Irish ethnicity, had never lived in a country outside Western Europe/USA/Canada, and indicated English as their first language. Japanese participants (*M* = 21.70 years, *SD* = 2.77 years) were born and raised in Japan, were of Japanese ethnicity, had never lived in a country outside East Asia, and indicated Japanese as their first language. All participants had normal or corrected-to-normal vision and hearing. The study lasted approximately 30 minutes and each participant received £8 (London) or ¥1000 (Kyoto) for their time in line with departmental regulations. The study was approved locally by the ethics committees of the Department of Psychological Sciences, Birkbeck, University of London, and the Department of Psychology, Kyoto University, and was conducted in accordance to the Declaration of Helsinki. Each participant provided written informed consent prior to the study.

### Apparatus

Eye movements were recorded using SMI eye tracking glasses (SMI ETG; SensoMotoric Instruments, Germany) at a sampling rate of 60 Hz. An integrated scene camera recorded the participant’s field-of-view (60° horizontally and 46° vertically), with two integrated eye cameras and infra-red LEDs used for binocular gaze tracking. Scene recordings were captured at 24 frames per second and at 1280 × 960 resolution.

### Procedure

**Figure 2:**
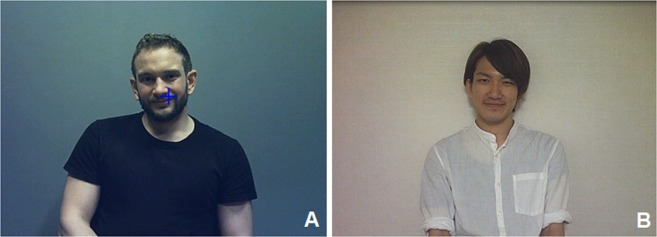
A participant’s view of the local research assistant. Snapshot taken from the head-mounted eye tracking footage during a dyadic interaction of a participant with the local research assistant in the UK (**A**) or in Japan (**B**).

The experimenter explained the study, presented the eye tracker, collected written informed consent, and asked the participant to fill out a demographic questionnaire. To ensure a naturalistic interaction, participants were informed that the content of their speech would not be used for analysis. The participants were also informed that the current study aimed to examine cultural differences in face perception, but it was not made explicit that face scanning strategies were being investigated. Informal interviews after the testing session confirmed that participants were not aware of the study aims. The participant was seated at a table opposite the local research assistant at approximately 1 metre distance. British/Irish participants interacted with a British research assistant (White-British ethnicity) in English, and Japanese individuals interacted with a Japanese research assistant (Japanese ethnicity) in Japanese (Fig. 2). Both research assistants were male and in their mid-20s.

To complete a three-point calibration procedure, the research assistant held the calibration object, which displayed concentric circles, in locations surrounding the face and asked the participant to fixate the circle centre. Participants were asked to fixate the calibration target an additional five times just before and after each experimental task for post-hoc data quality checks. The research assistant then explained the forthcoming task (described below), after which the experimenter left the room so that the dyadic interaction was not influenced by a third person. The experimenter returned after each task for re-calibration.

For the first task (*introduction)*, participants were asked to introduce themselves and were encouraged to speak for at least 30 seconds to obtain sufficient data for analysis. Participants were informed that they could mention their name, occupation, or hobbies, but that they were free to talk about anything as long as they were comfortable with sharing the personal details with their conversational partner. The research assistants were also asked to introduce themselves, and the participant and research assistant were free to have a conversation afterwards. The second task (*20 Questions)* consisted of two rounds of a guessing game in which one participant thought of an object while the other asked up to 20 questions to guess the object. Only ‘*Yes*’, ‘*No*’ or ‘*I don*’*t know*’ were permitted answers. An additional round of *20 Questions* was played if the object was guessed correctly before reaching 10 questions. Given that *20 Questions* has a clear structure, this game was included to facilitate a naturalistic interaction, but face scanning strategies were not analysed for this task since participants mostly averted their gaze away from the face. In the third task (*storytelling*), the participant and research assistant each picked a coin from the table, looked at the year shown on the coin, and told the other person about a personal event or experience that happened in that year. As with the introduction task, participants were asked to talk for at least 30 seconds. After the third task, the experimenter stopped the recording. For all tasks, the same experimenter instructed both the British and Japanese research assistant to control for content and duration of speech, which was kept constant across participants, and as similar as possible between cultures, language differences permitting. For instance, the research assistants mentioned facts such as their name, home town, and occupation, and repeated the same stories for every participant (e.g., having worked in a certain profession). Both research assistants practised the content and duration of speech during the piloting stage until they were well-rehearsed. Finally, participants were asked to complete the Autism Quotient (AQ)^28^ and the self-report version of the Liebowitz Social Anxiety Scale (LSAS-SR)^29^. Both the AQ and LSAS were provided in the participant’s native language and served to examine the relationship between face scanning and autistic or social anxiety traits in the general population.

### Data pre-processing

#### Data quality

Given that ethnicity can affect data quality^56^, post-hoc calibration points were presented after calibration and at the end of the experimental task to ensure spatial accuracy. Gaze data was examined offline in *BeGaze* (Version 3.7; SensoMotoric Instruments, Germany) by overlaying gaze points onto scene recordings and checking whether the crosshair fell onto the post-hoc calibration targets. When a consistent linear offset was detected, recalibration was performed in *BeGaze* using the gaze data obtained after original calibration, and this was confirmed using the gaze data obtained at the end of the task.

#### Coding of speaking and listening periods

For each task, the start time of the speaking period was defined as the first frame that contained audible speech (from the speaker) and the end time was determined when a participant stopped speaking. The start and end times of the listening period were coded accordingly. Speech was cropped at 30 seconds per task to ensure that participants contributed a similar amount of data^22^. When an interruption occurred, the end time was counted as the last frame just preceding the interruption. If less than 20 seconds of data was coded prior to interruption, a second start and end time would be used, starting from the second sentence after the speaker resumed speaking. The start of the second sentence was chosen given that individuals typically avert their gaze away from the face immediately after the start of speech^57^. No third start/end times of speech were required or used.

#### Regions-of-interest coding

**Figure 3:**
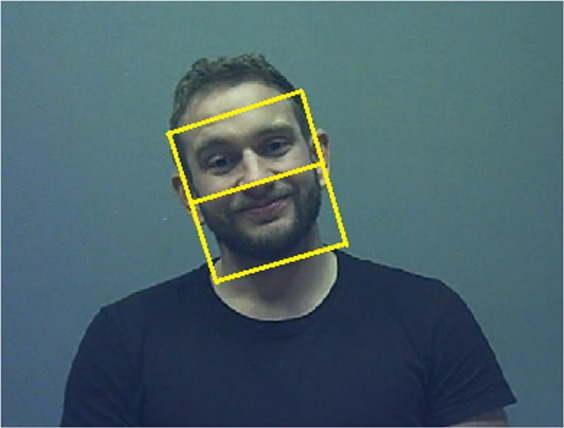
Regions-of-interest coding for the upper and lower face. A randomly selected frame from the scene recording showing the manually coded face region based on pre-defined guidelines, and the division of the face area into an upper and lower region.

A more detailed description is provided in the Supplementary Methods. The face was first detected automatically with a rectangular bounding box^58^, and the user was required to confirm detection to proceed to the next scene frame. If the user disagreed, the face would be marked up manually. For the current study, the following guidelines were used for the bounding box: the upper and bottom edges were located along the middle of the forehead and just underneath the chin, respectively, while the side edges were aligned with the sides of the face including a small margin (Fig. 3). The angular size of the face area measured approximately 11° (horizontal) × 14° (vertically). To track the face across frames, the Kanade-Lucas-Tomasi (KLT) algorithm^59,60^ was then applied using a minimum of 15 points to estimate the bounding box. To avoid a decline in tracking quality over time, automatic tracking terminated after 150 frames and returned to the face detection stage. For every frame, the coordinates of the four vertices of the bounding box (i.e., the face) were stored. The face area was further divided at the midline into an upper and a lower part as a proxy for the eye and mouth regions, respectively, in line with previous studies^24,61^. Each fixation was then classified by checking whether its coordinates fell within the upper or lower face region.

#### Gaze density maps

Unlike in screen-based paradigms, head-mounted eye tracking data cannot simply be collapsed across time and participants given that the position, size, and angle of the face changes with every frame. We developed a novel data-driven method for head-mounted eye tracking data that can also statistically analyse gaze density maps (see Supplementary Methods for a detailed description). Briefly, linear transformations were applied to re-map gaze points onto a normalised face template in a fully automatic fashion, allowing gaze data to be subsequently collapsed across time and participants. Each participant’s gaze density map was then spatially smoothed using a two-dimensional isotropic Gaussian kernel (2° width).

## Supplementary information

Supplementary Information.

Dataset 1.

## Data Availability

The raw datasets (scene videos) generated during the current study are not publicly available since they disclose personally identifiable information. However, all data analysed for this study are made available in the Supplementary Information.
