# Supplementary material for: Culture modulates face scanning during dyadic social interactions
Source: Sci Rep. 2020 Feb 6;10:1958. doi: 10.1038/s41598-020-58802-0 (PMC7005015; doi:10.1038/s41598-020-58802-0)
Supplement: Supplementary file 2 — Dataset 1. [file 41598_2020_58802_MOESM2_ESM.pdf]

Title: Culture modulates face scanning during dyadic social interactions

Authors: Jennifer X. Haensel*, Matthew Danvers, Mitsuhiko Ishikawa, Shoji Itakura, Raffaele Tucciarelli, Tim J. Smith, and Atsushi Senju

Supplementary Methods

The following provides a detailed description of the semi-automatic face detection and tracking processes and subsequent gaze classification. The methods for data-driven analysis of head-mounted eye tracking data are also presented.

Semi-automatic gaze classification of head-mounted eye tracking data

Face detection

Faces were located using the Viola-Jones detector[1] (Computer Vision System Toolbox, MATLAB R2015a, MathWorks). Face detection was visualised using a rectangular bounding box, and the user was required to confirm detection performance to proceed to the next scene frame. If the user disagreed, the face would be marked up manually by either dragging a rectangular box (for non-tilted faces) or marking four corner points to create a rectangular-like polygon (for tilted faces). Once the user confirmed that the bounding box accurately surrounded the face region, the coordinates of the four vertices were stored and the script proceeded to the next frame. For the current study, the following guidelines were used for the bounding box: the upper and bottom edges were located along the middle of the forehead and just underneath the chin, respectively, while the side edges were aligned with the sides of the face including a small margin. Alternatively, the user was able to skip the frame when no face was present. The user was also allowed to indicate the maximum number of frames that should be manually skipped (here the number was set to 2 frames) before an interactive video player was presented so that the user could more easily fast-forward to the next frame that contained a face.

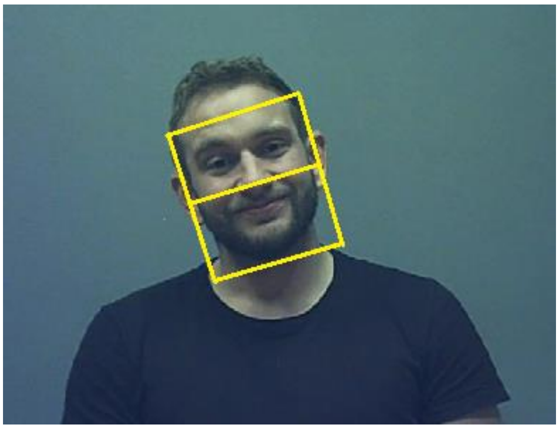

Supplementary Figure S1: Regions-of-interest coding for the upper and lower face. A randomly selected frame from the scene recording showing the manually coded face region based on pre-defined guidelines and the division of the face area into an upper and lower region.

Face tracking

Detecting the face in each frame is computationally inefficient, and feature tracking represents a superior approach. The Kanade-Lucas-Tomasi (KLT) algorithm[2,3] was applied to track the face using an adaptive window that changed the position, size, and angle of the bounding box in line with the face region. A video player visualised tracking behaviour and the four coordinates of the vertices of the bounding box were stored for each frame. For KLT

face tracking, the user was required to set the minimum number of feature points (*threshold*) that are used to estimate the bounding box. Although only very few points are typically required (e.g., 5 points), the threshold was increased to 15 points for the current study given that spatial accuracy and precision of face regions were crucial to investigate scanning behaviour. In addition, the user was required to specify the maximum number of frames to be processed to avoid a decline in tracking quality over time since points can be lost across frames. For the present study, a maximum of 150 frames were processed before the script returned to the face detection stage. Furthermore, a pushbutton was included to manually trigger the return to the face detection stage at any time in case the bounding box location could no longer be estimated accurately using the automatic algorithm. The flowchart in Supplementary Figure S2 summarises the face detection and tracking process.

Data extraction

The location of the face region was now known for every frame and given by the edge coordinates of the bounding box. The face area was subdivided into an upper and a lower part as a proxy for the eye and mouth regions, respectively. This was done by splitting the bounding box at the midline (Supplementary Figure S1). The eye tracking data was loaded into MATLAB to extract the *x*- and *y*-coordinates of the gaze points, and each gaze point was associated with its corresponding scene frame. The gaze point was classified by checking whether its coordinates fell within the upper or lower face region (using the `inpolygon` function). For each participant, a binary event timeline was created. An entry was coded ‘1’ if the gaze point fell within the lower/upper face, and ‘0’ if not. A speech timeline was added to annotate periods as listening (coded ‘0’) or speaking (coded ‘1’); this information was manually coded offline. Finally, an additional timeline indicated whether the gaze point was associated with a fixation (coded ‘1’) or not (coded ‘0’).

Coding performance

Manual checks were performed for 20% of data (10% per cultural group) collected for a separate study with the same paradigm. The mean accuracy was 99.02% (*SD* = 1.37%) for the upper face and 99.35% (*SD* = 0.97%) for the lower face. To code the upper and lower face and non-face regions for 1 minute of recording time, the semi-automatic method required 5 minutes and 16 seconds. Using a fully manual approach, gaze annotation took 11 minutes and 29 seconds (i.e., more than double the time).

Data-driven analysis of head-mounted eye tracking data

For screen-based eye tracking data, *iMap*[4,5] represents a data-driven method that aggregates gaze data across time and stimuli to produce density maps. Head-mounted eye tracking data, however, cannot simply be collapsed given that the position, size, and angle of the face changes with every frame. We applied linear transformations to re-map gaze points onto a normalised face template in a fully automatic fashion. *Monte Carlo permutation testing* (also named *approximate permutation test* or *random permutation test*)[6] was then used to identify cultural differences in gaze clustering.

Data extraction

To collapse gaze points across time and participants, the original absolute gaze coordinates that fell within the face region were re-expressed as relative coordinates with respect to the bounding box (rather than the scene frame), making them independent of the location, size, or angle of the face. This was achieved using the following steps:

(1) Non-rectangular bounding boxes (for non-tilted, four-point polygonic shapes) were first transformed by fitting a minimally bounding rectangle around the four vertices.

(2) Each bounding box and its corresponding gaze points were then rotated such that the top and bottom edges were aligned in parallel with the *x*-axis of the scene frame, i.e. such that the face was no longer tilted. Rotations were not required to be performed

around a specific point. The angle α between the bottom edge of the bounding box and the *x*-axis was first computed to set up a rotation matrix *R*,

$$R = \begin{bmatrix} \cos(-\alpha) & -\sin(-\alpha) \\ \sin(-\alpha) & \cos(-\alpha) \end{bmatrix}.$$

To perform clockwise rotations, the angle α is negative in this rotation matrix. *R* was then used to rotate the bounding box and the original gaze coordinates to obtain the new coordinates of each shifted vertex (v_x', v_y') and the new gaze coordinate (x', y'),

$$\begin{bmatrix} v_x' \\ v_y' \end{bmatrix} = R \begin{bmatrix} v_x \\ v_y \end{bmatrix}; \begin{bmatrix} x' \\ y' \end{bmatrix} = R \begin{bmatrix} x \\ y \end{bmatrix}.$$

(3) The new gaze coordinates were then expressed relative to the rotated bounding box by setting its vertices to v_1 = (-1, -1), v_2 = (1,-1), v_3 = (1,1), and v_4 = (-1,1), i.e. the origin represented the centre of the face (nose tip).

(4) For every participant and for each condition, a grid was set up to map all relative gaze coordinates into a unified coordinate space. For this study, a 100 x 100 grid was used with the same vertices as the bounding box, i.e., v_1 = (-1,-1), v_2 = (1,-1), v_3 = (1,1), and v_4 = (-1,1).

(5) Each relative gaze coordinate was then mapped into the grid by finding its location within the grid and filling the corresponding entry. This represented the density maps with gaze collapsed across time.

(6) The density maps were smoothed to consider both measurement error and that foveal visual attention occurs not only at the precise coordinate position but is distributed within 1.5° to 2° visual angle[7]. In this study, smoothing was performed using a two-dimensional isotropic Gaussian kernel, with a kernel width corresponding to 2° (using `imgaussfilt`).

Statistical analysis

Comparing each pixel individually when contrasting 100px *x* 100px maps would result in 10,000 independent *t*-tests and introduce the *multiple comparison problem.* If the alpha-level is set to 0.05 for a single *t*-test, this would give 500px flagged as significant by chance. To adjust the alpha-level from a local scale (i.e., a single pixel) to a global scale (i.e., the entire map), several approaches are available. The Bonferroni correction method approximates an adjusted significance threshold by dividing the alpha-value by the number of tests (i.e., 0.05 / 10,000 = 0.000005 in the above example). This threshold, however, is too conservative due to the notion of *spatial correlation*. Given the spatial smoothing, gaze points are to an extent spatially dependent. The Bonferroni correction method, however, assumes independence between pixels, such that the adjusted threshold is overly conservative. An alternative method is based on Random Field Theory (RFT)[8,9], which also provided the framework for *iMap*[4]. The smoothness underlying the activation maps is estimated, and the Euler characteristic (the number of clusters or "blobs" after thresholding)[10] is determined at varying thresholds. The threshold at which 5% (0.05 alpha-level) of equivalent statistical maps would occur under the null hypothesis can then be computed. RFT requires a Gaussian distribution and sufficient smoothness, and represents a powerful method when assumptions are met. However, RFT may produce unreliable results when data is not normally distributed or for paradigms with a low number of participants since maps may not necessarily be sufficiently smooth[10].

Another approach – and the one chosen here – is non-parametric permutation testing[6], which does not require data to be normally distributed, and has previously been implemented in two screen-based studies[11,12]. In contrast to previous studies[11,12], however, we applied a cluster-based approach to correct for multiple comparisons (as opposed to, e.g., FDR). Permutation testing uses the observed data itself to generate a null distribution that describes a gaze distribution that is entirely random. This is obtained by exchanging the data across conditions or groups in all possible arrangements to compute the frequency

distribution of test statistics (e.g., t-score). Consider a between-subject design with Participants A and B in one group, and Participants C and D in another group. By shuffling participants into all possible combinations, test statistics are calculated for AB (Group 1) vs CD (Group 2), AC (Group 1) vs BD (Group 2), and AD (Group 1) vs BC (Group 2) to obtain the null distribution, i.e. the distribution of test statistics if group allocations were random. Naturally, these permutations are typically conducted on data sets with larger participant numbers, and computing all possible permutations is time-consuming and computationally demanding. The *Monte Carlo method*[13] can approximate the null distribution by running many permutations – typically in the order of several thousand iterations. Once the null distribution is computed, the proportion of test statistics that result in larger values than the observed statistic (the *Monte Carlo significance probability*) can be calculated. To obtain significant differences, this proportion should be minimal (e.g., less than 5%, or $p < 0.05$). Permutation testing only assumes *exchangeability*[6] – i.e. data needs to be exchangeable across conditions or groups – and this assumption is met when exchanging data sets from different participants.

The Monte Carlo permutation test was implemented in MATLAB using the *CoSMoMVPA* toolbox[14] and *FieldTrip* toolbox[15]. The statistical analysis involved *cluster-based* permutation tests, whereby a clustering procedure was applied to the original data set and to each permutated data set that was obtained by swapping participants between the cultural groups. Specifically, the clustering procedure involved identifying neighbouring pixels if their test criterion was greater than the critical value t_{crit} associated with a specified p-value threshold. This threshold was required to be set by the user, and a moderately strict threshold of 0.01 was chosen for the current study. To examine which clusters in the original map were significant, a cluster statistic was selected and used as comparison with each permuted map. We chose the size of the cluster as the statistic for the present analyses. For every iteration, the statistic of each cluster in the original map was compared against that in the permuted map. After all iterations were performed (here the number of iterations was set to 10,000), the Monte Carlo significance probability was calculated; in other words, the proportion of test statistics that resulted in a larger value than the actual observed statistic of the original cluster was obtained. If this only occurred very few times, i.e. less than 5% (0.05) of times, this cluster was flagged as significant.

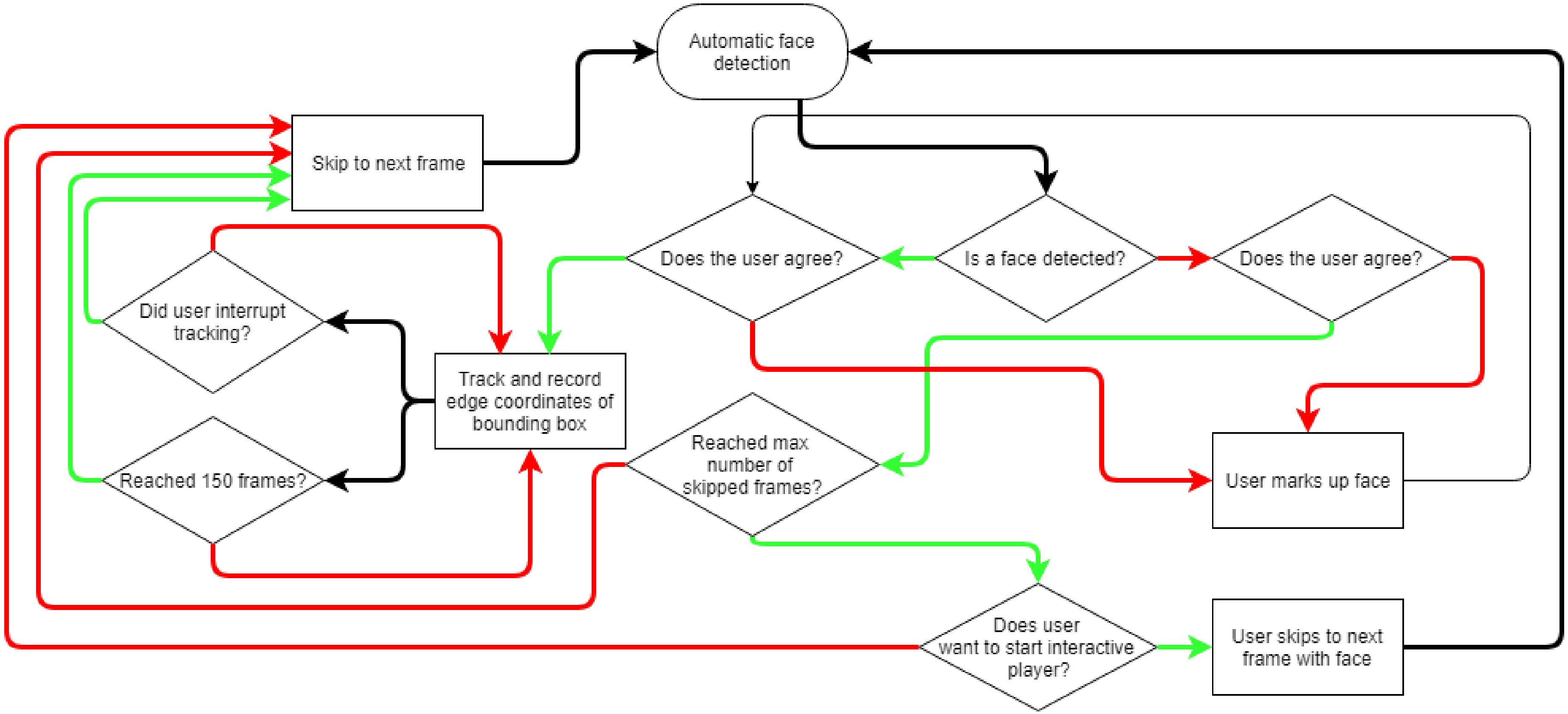

Supplementary Figure S2: Flowchart of the semi-automatic face detection and tracking process. Green and red lines indicate ‘yes’ and ‘no’ responses, respectively

Supplementary Analysis

To strengthen the current study interpretations, the following briefly presents the methods and results of a subset of the data obtained from a separate screen-based face scanning study[16]. The screen-based study aimed to examine cultural differences in face scanning and also included other cognitive tasks and tested infant and adult age groups. Here, only relevant face scanning data collected from the adult sample is presented. The sample consisted of the majority of participants who also took part in the present dyadic interaction study, with the results revealing cultural differences in face scanning. This suggests that the observed group differences in face scanning in the current dyadic interaction study are unlikely to be attributed solely to the local research assistant's individual-specific behaviour.

<u>Methods</u>
Thirty-one British (16 female) and 30 Japanese adults (17 female) participated in the screen-based study, of which 24 British and 26 Japanese participants also took part in the current dyadic interaction study. The same participant criteria as in the dyadic interaction study were applied.

<u>Apparatus</u>
A Tobii TX300 eye tracker (Tobii Technology, Sweden) was used to record eye movements at a sampling rate of 120 Hz. Face stimuli were presented on a 23" monitor.

<u>Procedure</u>
Participants sat in front of the monitor at 65cm distance. A five-point calibration was conducted prior to the start of the free-viewing experiment. Face stimuli were interleaved with other cognitive tasks, and presented in either static conditions (image of a face) or dynamic conditions (video of a face speaking the syllables *do re mi fa sol la ti do*). In each condition, every participant was shown four female face identities with a neutral facial expression (two faces of White-British ethnicity and two of Japanese ethnicity). Each face trial started with a gaze-contingent central stimulus before the face stimulus (measuring 16.5° in height and 12.0° in width) was displayed for 18 seconds in colour at 1920 x 1080 resolution (see Supplementary Figure 3 for an example). Sound was muted and replaced with instrumental music. Face identities were never repeated and were matched in location and speech timings.

<u>Results</u>
Given that the original study included eye movement data from both infants and adults, fixations were obtained using GraFIX, a semi-automatic tool designed to parse eye-tracking data of varying quality[17]. Regions-of-interest (ROI) included the eyes, bridge, nose, and mouth (see Supplementary Figure S3), and fixation time proportional to inner face fixation time was obtained for each ROI.

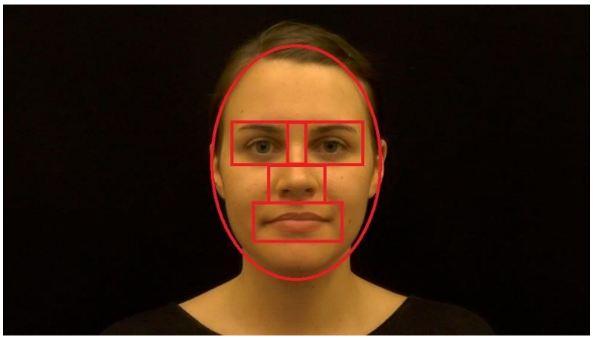

Supplementary Figure S3: Regions-of-interest superimposed onto a face.

For the purpose of this Supplementary Analysis, a three-way ANOVA was conducted with factors Group (British, Japanese), Face Ethnicity (White-British, Japanese), and ROI (eyes, bridge, nose, mouth), separately for static and dynamic faces. Greenhouse-Geisser estimates were used when the sphericity assumption was not met.

Static faces

A main effect of ROI was revealed ($F(3, 177) = 111.40$, $p < 0.001$, $\eta_p^2 = 0.654$), showing that scanning patterns were not homogeneous across facial features. The ROI x Face Ethnicity interaction was also significant ($F(3, 177) = 4.75$, $p = 0.003$, $\eta_p^2 = 0.074$). A follow-up analysis using paired-samples *t*-tests, collapsed across cultural groups, revealed more eye scanning and less bridge scanning of White-British faces compared to Japanese faces (eyes: $t(60) = -2.46$, $p = 0.017$, Cohen's $d = 0.221$; bridge: $t(60) = 2.52$, $p = 0.014$, Cohen's $d = 0.380$). No significant differences were observed for the nose ($t(60) = 0.83$, $p = 0.409$, Cohen's $d = 0.065$) or mouth ($t(60) = -0.77$, $p = 0.444$, Cohen's $d = 0.085$). The relevant effects for the present analysis, however, are those including both ROI and Group as factors. A ROI x Group interaction was found ($F(3, 177) = 2.79$, $p = 0.042$, $\eta_p^2 = 0.045$), suggesting that scanning patterns differed between the cultural groups. No other effects were significant (Face Ethnicity: $F(1, 59) = 0.47$, $p = 0.495$, $\eta_p^2 = 0.008$; Group: $F(1, 59) = 0.19$, $p = 0.663$, $\eta_p^2 = 0.003$; Face Ethnicity x Group: $F(1, 59) = 0.15$, $p = 0.698$, $\eta_p^2 = 0.003$; Face Ethnicity x ROI x Group: $F(3, 177) = 1.16$, $p = 0.328$, $\eta_p^2 = 0.019$). To follow up the ROI x Group interaction, the fixation data was collapsed across the two levels of Face Ethnicity, and independent *t*-tests were conducted to compare cultural groups for each ROI. The findings revealed no significant cultural differences in eye scanning (British: $M = 0.49$, $SD = 0.14$; Japanese: $M = 0.48$, $SD = 0.22$; $t(59) = 0.13$, $p = 0.902$, *Cohen's* $d = 0.032$), bridge scanning (British: $M = 0.08$, $SD = 0.06$; Japanese: $M = 0.10$, $SD = 0.07$; $t(59) = -1.39$, $p = 0.170$, *Cohen's* $d = 0.379$), or nose scanning (British: $M = 0.15$, $SD = 0.10$; Japanese: $M = 0.19$, $SD = 0.18$; $t(59) = -1.19$, $p = 0.240$, *Cohen's* $d = 0.306$), but demonstrated that British adults scanned the mouth significantly more than Japanese participants (British: $M = 0.13$, $SD = 0.05$; Japanese: $M = 0.09$, $SD = 0.04$; $t(59) = 4.63$, $p < 0.001$, *Cohen's* $d = 1.180$).

Dynamic faces

Main effects of ROI ($F(1.99, 117.09) = 50.44$, $p < 0.001$, $\eta_p^2 = 0.461$) and Face Ethnicity ($F(1, 59) = 7.80$, $p = 0.007$, $\eta_p^2 = 0.117$) were revealed. Crucially, a ROI x Group interaction was found ($F(1.99, 117.09) = 3.42$, $p = 0.036$, $\eta_p^2 = 0.055$). No other effects were significant (Group: $F(1, 59) = 2.17$, $p = 0.146$, $\eta_p^2 = 0.035$; ROI x Face Ethnicity: $F(1.67, 98.71) = 0.29$, $p = 0.712$, $\eta_p^2 = 0.005$; Face Ethnicity x Group: $F(1, 59) = 2.81$, $p = 0.099$, $\eta_p^2 = 0.045$; Face Ethnicity x ROI x Group: $F(1.67, 98.71) = 0.09$, $p = 0.885$, $\eta_p^2 = 0.001$). As with the static faces, independent *t*-tests were conducted to compare cultural groups at each level of ROI, collapsed across Face Ethnicity. No cultural differences were found for eye scanning (British: $M = 0.24$, $SD = 0.13$; Japanese: $M = 0.21$, $SD = 0.16$; $t(59) = 0.95$, $p = 0.346$, *Cohen's* $d = 0.263$) or bridge scanning (British: $M = 0.05$, $SD = 0.06$; Japanese: $M = 0.07$, $SD = 0.08$; $t(59) = -1.07$, $p = 0.290$, *Cohen's* $d = 0.336$). However, Japanese adults scanned the nose significantly more than British participants (British: $M = 0.14$, $SD = 0.09$; Japanese: $M = 0.23$, $SD = 0.17$; $t(59) = -2.54$, $p = 0.014$, *Cohen's* $d = 0.647$), while the British group tended to scan the mouth region more (British: $M = 0.49$, $SD = 0.18$; Japanese: $M = 0.39$, $SD = 0.24$; $t(59) = 1.88$, $p = 0.065$, *Cohen's* $d = 0.481$).

References

1. Viola, P. & Jones, M. Rapid object detection using a boosted cascade of simple features. in *Proceedings of the 2001 IEEE Computer Society Conference on Computer Vision and Pattern Recognition. CVPR 2001* **1**, I-511-I–518 (IEEE Comput. Soc, 2001).
2. Lucas, B. D. & Kanade, T. An Iterative Image Registration Technique with an Application to Stereo Vision. *Proc. 7th Int. Jt. Conf. Artif. Intell.* 674–679 (1981).
3. Tomasi, C. & Kanade, T. Detection and Tracking of Point Features. (1991).
4. Caldara, R. & Miellet, S. iMap: a novel method for statistical fixation mapping of eye movement data. *Behav. Res. Methods* **43**, 864–878 (2011).
5. Lao, J., Miellet, S., Pernet, C., Sokhn, N. & Caldara, R. iMap4: An open source toolbox for the statistical fixation mapping of eye movement data with linear mixed modeling. *Behav. Res. Methods* **49**, 559–575 (2017).
6. Nichols, T. E. & Holmes, A. P. Nonparametric permutation tests for functional neuroimaging: A primer with examples. *Hum. Brain Mapp.* **15**, 1–25 (2002).
7. Holmqvist, K. *et al. Eye Tracking: A comprehensive guide to methods and measures*. (Oxford University Press, 2011).
8. Adler, R. J. *The Geometry of Random Fields*. (Wiley, 1981).
9. Worsley, K. J., Marrett, S., Neelin, P., Friston, K. J. & Evans, A. C. A Unified Statistical Approach for Determining Significant Signals in Images of Cerebral Activation. *Hum. Brain Mapp.* **4**, 58–73 (1996).
10. Brett, M., Penny, W. & Kiebel, S. An Introduction to Random Field Theory. *Hum. Brain Funct.* **2**, 1–23 (2003).
11. Arizpe, J., Kravitz, D. J., Walsh, V., Yovel, G. & Baker, C. I. Differences in Looking at Own- and Other-Race Faces Are Subtle and Analysis-Dependent: An Account of Discrepant Reports. *PLoS ONE* **11**, e0148253 (2016).
12. Arizpe, J., Kravitz, D. J., Yovel, G. & Baker, C. I. Start Position Strongly Influences Fixation Patterns during Face Processing: Difficulties with Eye Movements as a Measure of Information Use. *PLoS ONE* **7**, e31106 (2012).
13. Manly, B. F. J. *Randomization, bootstrap and Monte Carlo methods in biology*. (Chapman & Hall, 1997).
14. Oosterhof, N. N., Connolly, A. C. & Haxby, J. V. CoSMoMVPA: Multi-Modal Multivariate Pattern Analysis of Neuroimaging Data in Matlab/GNU Octave. *Front. Neuroinformatics* **10**, 1–27 (2016).
15. Oostenveld, R., Fries, P., Maris, E. & Schoffelen, J.-M. FieldTrip: Open Source Software for Advanced Analysis of MEG, EEG, and Invasive Electrophysiological Data. *Comput. Intell. Neurosci.* **2011**, 1–9 (2011).
16. Haensel, J. X., Ishikawa, M., Itakura, S., Smith, T. J. & Senju, A. Culture modulates face scanning across development. (under review).
17. Saez de Urabain, I. R., Johnson, M. H. & Smith, T. J. GraFIX: A semiautomatic approach for parsing low- and high-quality eye-tracking data. *Behav. Res. Methods* **47**, 53–72 (2015).
